# Ocimum flavone Orientin as a countermeasure for thrombocytopenia

**DOI:** 10.1038/s41598-018-23419-x

**Published:** 2018-03-22

**Authors:** Marshleen Yadav, Feifei Song, Jason Huang, Arnab Chakravarti, Naduparambil K. Jacob

**Affiliations:** Department of Radiation Oncology, Comprehensive Cancer Center, The Ohio State University, Columbus, OH 43210 USA

## Abstract

Thrombocytopenia or chronic depletion of platelets in blood, could create life-threatening conditions in patients who receive aggressive systemic radiation and chemotherapy. Currently there are no approved agents for the rapid treatment of thrombocytopenia. In the present study, we demonstrate that administration of Orientin, a glycosidic flavonoid or dietary administration of Orientin containing Tulsi (Holy Basil) leaves, results in a significant increase in circulating platelets in a clinically relevant mouse model. No noticeable effects were observed on red blood cells, white blood cells or other hematologic parameters in treated animals indicating that Orientin specificity enhances platelet formation. The gene expression and immunophenotyping of bone marrow revealed that Orientin stimulates megakaryopoiesis specific transcriptional program. A significant increase in colony formation in bone marrow cells from Orientin pretreated mice further complemented the effect of Orientin on progenitor cells. The *ex-vivo* differentiation of irradiated human peripheral blood CD34^+^ stem cells demonstrated stimulatory effects of Orientin on megakaryocyte erythrocyte progenitors (MEP). The results show that Orientin, a non-toxic readily available natural product can counter platelet imbalances. Thrombocytopenia also develop as a consequence of multiple hematologic malignancies and side effects of treatments. Dietary supplementation of Orientin containing phytochemicals could be effective as countermeasures and viable therapeutics.

## Introduction

Thrombocytopenia, the lowering of platelet count, is a major side effect in cancer patients who receive total body irradiation (TBI) and systemic chemotherapy^1,2^. Moreover, thrombocytopenia is one of the leading causes of bone marrow transplant associated deaths^3,4^. As a result, platelet infusion has become a clinical practice in more than 30% of bone marrow transplant patients^5,6^. Thrombocytopenia is also common in patients who receive cisplatin and other cytotoxic agents to treat solid tumors as well, although the acute effects here are less severe in comparison to that in transplant patients who undergo radiation and chemo-based conditioning.

Normal tissue toxicity is a major concern in cancer patients receiving aggressive treatment, manifested as acute radiation syndromes (ARS), affecting hematopoietic, gastrointestinal and cerebrovascular systems. Countermeasures are available for clinical management of neutropenia which include granulocyte-colony stimulating factor, G-CSF (Neupogen/Filgrastim)^7^ but currently there are no agents that received regulatory approval for rapid treatment of radiation or chemotherapy induced thrombocytopenia. Earlier studies have shown that countermeasures such as recombinant thrombopoietin (rhTPO) and PEGylated recombinant megakaryocyte growth and development factor (PEG-rHuMGDF) trigger immunogenicity in patients and volunteers^8–10^. Despite facilitating the platelet collection in donors and providing some benefits in non-ablative chemotherapy patients, both molecules failed to achieve platelet recovery in acute leukemia patients who received myeloablative regimens^2^. Also, current second generation TPO receptor agonists Romiplostim and Eltromopag have not been optimized yet to treat thrombocytopenia in cancer patients^11^. Therefore, there is a need for development of non-toxic, non-immunogenic compounds that could enhance the grafting efficiency without mitigating the cytotoxic and ablative effects of radiation on targeted cell population such as lymphocytes and their progenitors.

In the present study, we sought to investigate the effect of Orientin, a glucoside of luteolin, in preventing (administrated before radiation) or mitigating (administered after radiation) some of the toxic effects of radiation by enhancing platelets production in a mouse model. Orientin was originally isolated from Indian “Holy Basil” *Ocimum sp*. and was shown to provide survival benefits in mice after total body irradiation^12^. Inherent dose-dependent anti-oxidant property shown by flavonoids was proposed as a general mechanism of protection against free-radicals. Studies have shown that Orientin is able to provide survival benefit in mice at a very low dose of 50 µg/kg body weight, indicating that the radio-protective effect of this agent is beyond its systemic anti-oxidant effect^12–16^.

Our *in vivo* mouse model studies demonstrated an accelerated platelet recovery in peripheral blood with concomitant increase in bone marrow megakaryocytes in Orientin treated irradiated mice compared to that in irradiation alone group. Gene expression profile of bone marrow cells from Orientin treated irradiated group showed modulation of genes involved in megakaryocyte erythrocyte progenitor (MEP) lineage contributing to enhanced platelet recovery complementing the *in vivo* readout. Importantly, Orientin effects were found to be restricted to platelet forming cells without mitigating the cytotoxic effects of radiation on lymphocytes, underscoring a clinically relevant protective benefit on specific lineage.

## Results

### Administration of Orientin improves platelet count in mouse peripheral blood

**Figure 1:**
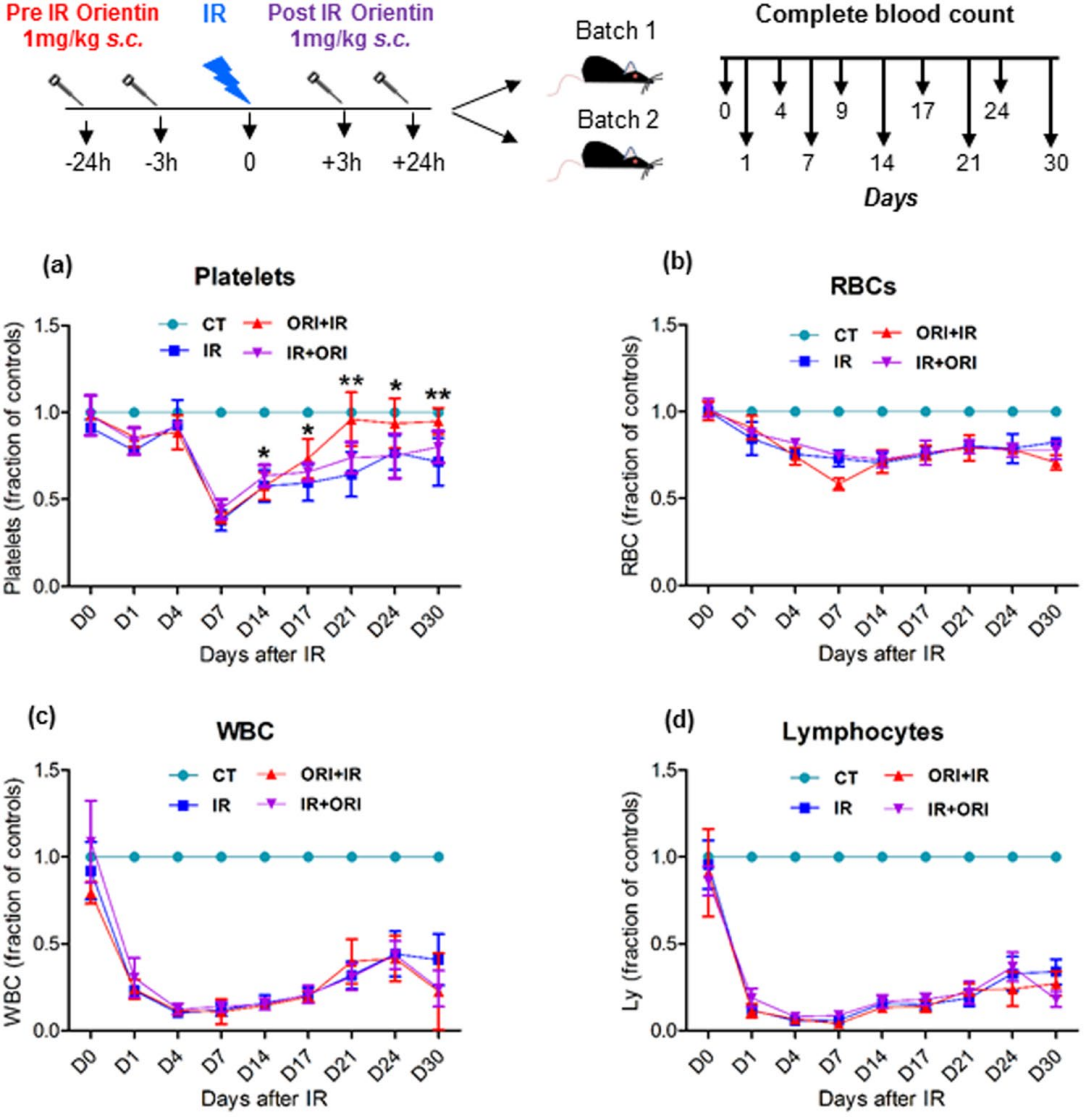
Orientin administration counteracts radiation induced thrombocytopenia in animal model. Complete Blood Count (CBC) reveals enhanced platelet reconstitution in Orientin pre and post treatment group (1 mg/kg Orientin via subcutaneous route 24 h and 3 h before radiation) at time points after 6 Gy partially ablative TBI (137Cs gamma rays) in comparison to irradiated controls. The difference noted were statistically significant at day 17, 21, 24 and 30 with *p < 0.05 and **p < 0.005 compared to irradiation alone group. All the cell counts were normalized with that of unirradiated and untreated controls (CT). Mice receiving Orientin treatment 3 h and 24 hr after 6 Gy IR also showed a nominal increase in platelets (**a**) at Day 21 and 24, however not statistically significant. Analysis of RBCs (**b**), WBCs (**c**) and Lymphocytes (**d**) revealed no significant differences between the groups. Three independent experiments were performed with at least 5 animals in each group and line graphs shown are mean ± SD of a representative experiment.

Earlier studies have shown that longitudinal analysis of blood parameters after sub-lethal irradiation in mice allows the evaluation of the effects of radioprotectors and radiomitigators^17^. In our animal TBI model, the platelets, erythrocytes, lymphocytes and other blood parameters were found depleted within 4 days of irradiation, reaching a nadir by day 7 in all irradiated groups (Fig. 1, Supplemental Table 1). However, post-nadir, we observed an early and gradual increase in platelets in ORI pretreated (1 mg/kg body weight via subcutaneous route) cohorts, upregulated to a significant level by day 17 (ORI + IR 540 ± 55 k/µL; *P* = 0.021 and IR + ORI 483 ± 38 k/µL) compared with IR alone group (438 ± 53 k/µL) (Fig. 1a). A sustained elevation in platelet count was observed in ORI pre-treated animals which peaked at day 21 (ORI + IR 677 ± 89 k/µL vs IR 439 ± 71 k/µL; *P* = 0.00051) to day 24 (ORI + IR 694 ± 65 k/µL vs IR 573 ± 23 k/µL; *P* = 0.039) and returned to baseline level by day 30 with no rebound thrombocytopenia. At all these time points, ORI + IR group exhibited approximately 40% difference in platelet count compared with IR alone cohort. Furthermore, we also observed an increase in plateletcrit (PCT, a measurement derived from platelet count and mean platelet volume) that is a commonly used clinical parameter for measuring thrombocytopenia (Supplementary Figure S1a). The platelet reconstitution was also observed in Orientin post-treated mice at indicated time points; however the effect was not as pronounced as observed in pretreatment. The levels of RBCs, WBCs and lymphocytes in peripheral blood were found similar in all irradiated cohorts at any of the tested time points, indicating that the benefit of Orientin administration was specific towards platelet recovery (Fig. 1b–d). We further investigated whether or not Orientin exhibits any effect on other myeloid and lymphoid cells by immunophenotyping of CD3, CD220, and CD11b and Ly6G markers in peripheral blood of IR or IR + ORI pre/post treated mice post day 30 (Figure S1b). However, no significant differences were detected in proportion of any of the measured parameters (granulocytes/monocyte/macrophages, T and B cells) between the cohorts, further confirming the specificity towards platelet forming cells.

### Dietary administration of Tulsi (Holy Basil) leaves enhances the platelet count in peripheral blood

**Figure 2:**
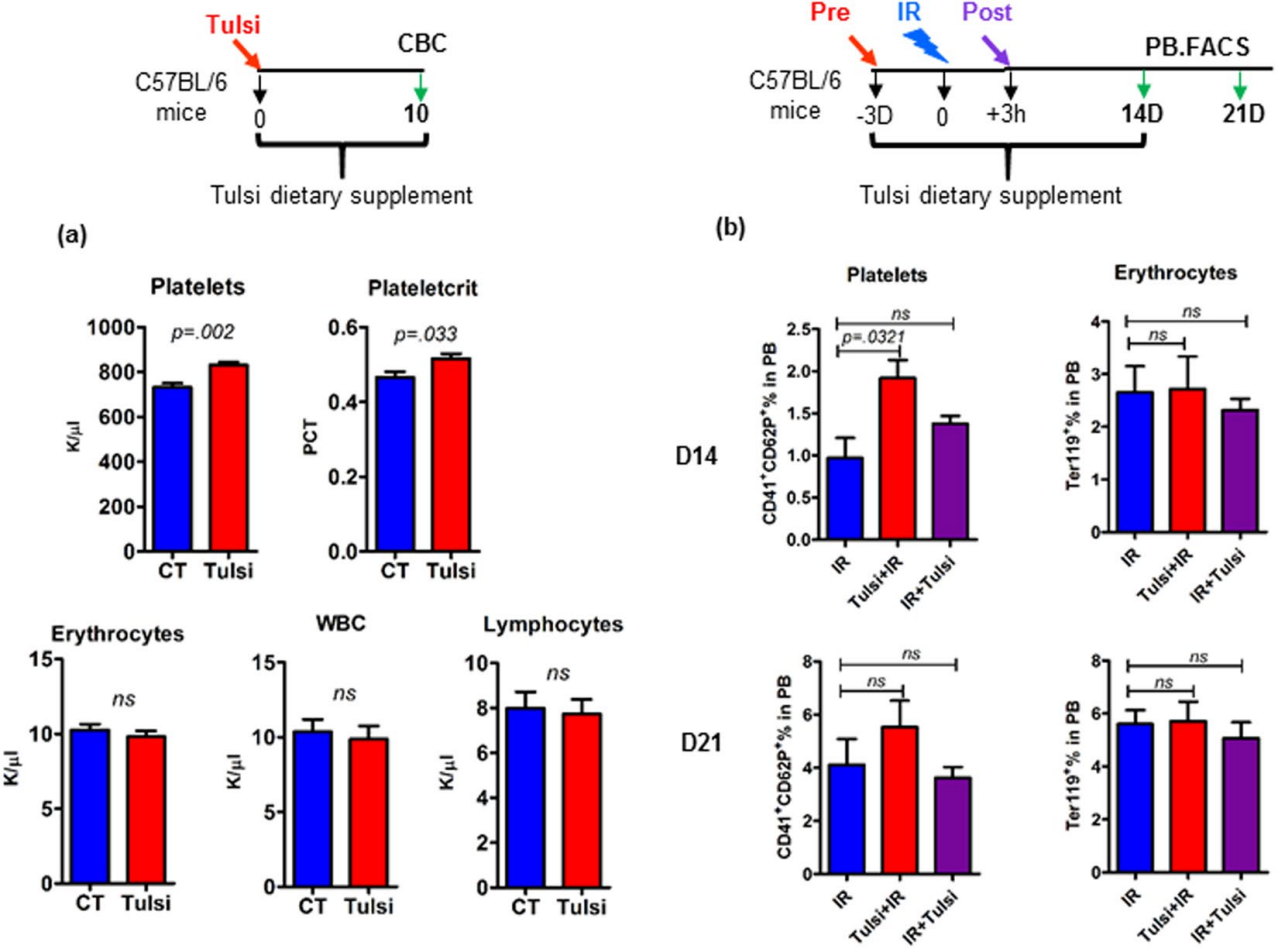
Dietary administration of Tulsi enhances peripheral blood platelet counts in animal model. (**a**) Feeding mice with Tulsi containing food (1% by weight) resulted in an increase specifically in platelet numbers and plateletcrit (PCT) as evaluated by complete blood count at Day 10 after starting dietary supplementation of Tulsi (**b**) FACS analysis showing percentage of platelets (CD41^+^CD62P^+^) and erythrocytes (Ter119^+^) in Tulsi pretreated or post treated versus irradiation alone groups; Tulsi + IR (pre), IR + Tulsi (post) and IR (6 Gy) alone groups. Bars are mean ± SD of n = 5 animals/treatment group.

In order to investigate the rapid translational potential of the novel finding, we further compared the blood parameters of animals after dietary supplementation of Tulsi (“Holy basil”) leaves, a natural source of Orientin^18^. Animals were fed with powdered diet containing dried Tulsi leaves (1% by weight) for ten days (time point slightly higher than the platelet cycle in mice)^19^ and blood parameters were compared by complete blood count. A statistically significant increase in platelet number was observed in animals fed with Tulsi leaf powder containing rodent chow when compared with that of those fed with regular chow (Tulsi 831 ± 30 k/µL *vs* CT 732 ± 38 k/µL; *P* = 0.002) with no significant changes in any other blood parameters (Fig. 2a, supplemental Table 2). Consistent with platelet count, a statistically significant increase in PCT was noticed in Tulsi treated mice (Fig. 2a). The observed beneficial effects of dietary administration of Tulsi on platelets was also visible in Tulsi pretreated and irradiated mice (Tulsi + 6 Gy IR, sub-lethal, partially ablative) where a 2 and 1.4 fold increase in platelet surface markers CD41^+^CD62P^+^ was detected at day 14 (*P* = 0.0321) and 21 respectively compared with IR alone group with no alteration in erythrocyte marker Ter119^+^ at any of the measured time point (Fig. 2b). This data further confirms that the intake of relatively low and physiologically achievable level of Orientin by dietary consumption of Basil leaves can enhance platelet counts in peripheral blood, underscoring its utility for potential human use.

### Orientin rescues bone marrow megakaryocytes post radiation injury

**Figure 3:**
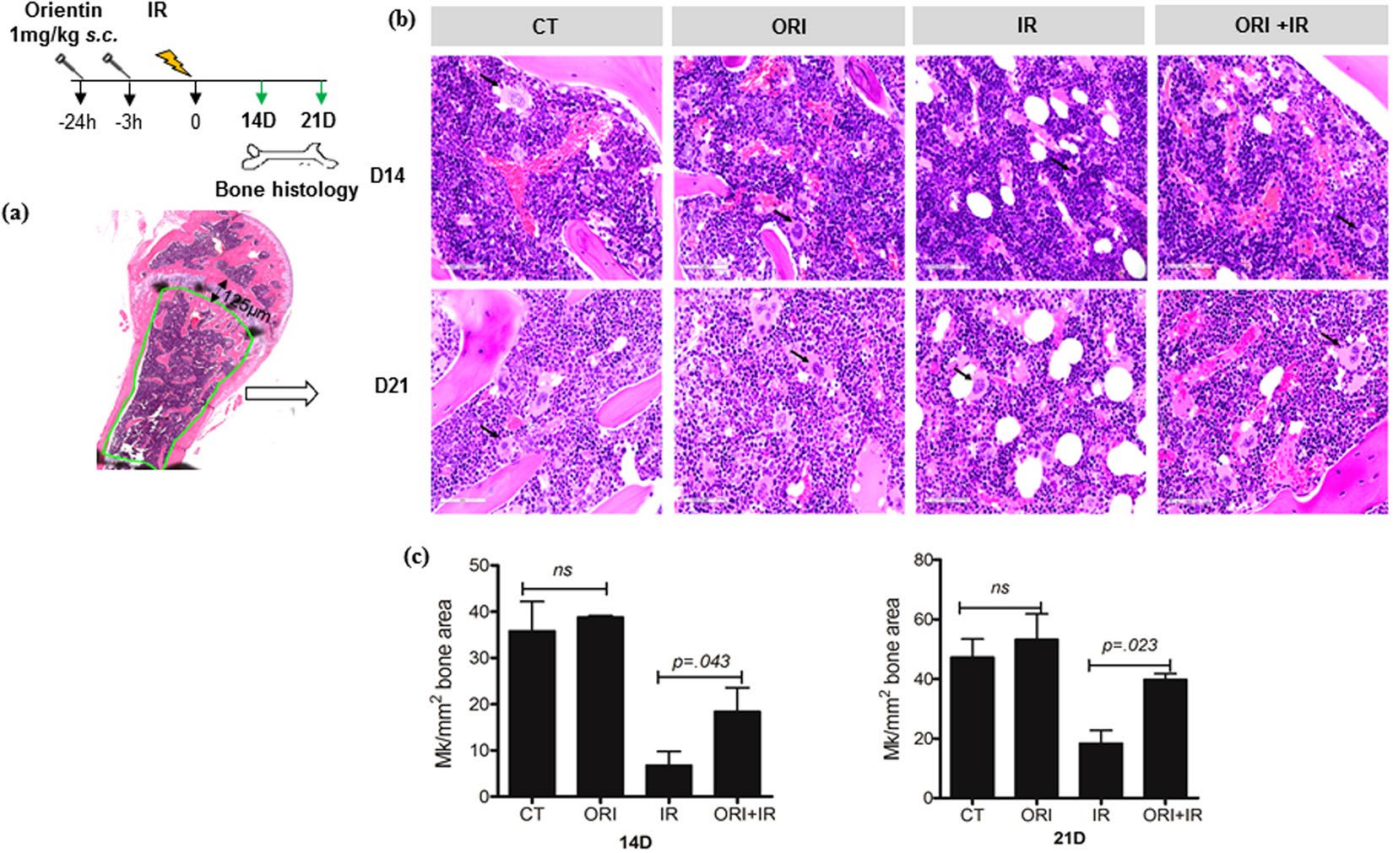
The bone histology shows an increased megakaryocyte number. MK scoring was done by a subjective analysis of three adjacent high power (60x) microscopic fields for each H&E stained bone section. (**a**) Megakaryocytes/2 mm^2^ tissue area was scored 125 µm away from the growth plate for a total area of 2 mm^2^. (**b**) Representative tissue sections depict effect of Orientin on megakaryocytes on day 21 post 6 Gy TBI in mice (C57BL/6 8–10 week old). Black arrow denotes MK cells. Scale bar 50 µm (**c**) MK count from two independent experiments scored after indicated days of 6 Gy exposure (n = 5 each group).

To determine whether or not the changes detected in peripheral blood corresponds to the level of megakaryocytes (MK) in the bone marrow, we performed bone histology of mice on day 14 and day 21 post irradiation, two time points where significant differences were detected in peripheral blood analyses. The H&E (Hematoxylin and Eosin) stained sterna bone sections revealed a marked depletion in overall marrow cellularity in all irradiated groups. Interestingly, Orientin treated mice (ORI + IR) exhibited almost 2 fold higher MK counts compared with IR alone group at measured time points (day 14; ORI + IR 18.4 ± 7.2/2 mm^2^ vs IR 6.77 ± 4.2/2 mm^2^ and day 21; ORI + IR 39 ± 2.77/2 mm^2^ vs IR 18 ± 6.1/2 mm^2^; *P* < 0.05) as shown in Fig. 3a–c. We also found an increase in MK cells in Orientin alone group (ORI) where the count was over 45 ± 5.6/2 mm^2^ area at both time points compared to that of untreated PBS controls (CT) (40 ± 8.9/2 mm^2^) although the difference was not statistically significant (Fig. 3c).

The enhanced platelet count and MK cells in Orientin treated animals prompted us to investigate cell population enriched in MK progenitors (CD41) in bone marrow (BM). Based on the evaluation of platelet depletion and reconstitution kinetics post irradiation, we selected two specific time points; day 9, as an early time point where emergence of marrow reconstitution is expected post IR injury and day 18, as a later time point where measurable immune readout of marrow is expected in peripheral blood. We segregated the cell population in three fractions using flow cytometry: c-Kit^+^CD41^−^ (hematopoietic progenitors), c-Kit^+^CD41^+^ (megakaryocytic progenitors), and c-Kit^−^CD41^+^ (megakaryocytes) cells (Figure S2a). At day 9 in ORI + IR group, c-Kit^+^CD41^−^ cells were found 1.6 fold higher (11.19 ± 1.73%; *P* = 0.045) compared to IR group (6.79 ± 1.34%). A modest (1.3 fold) increase in co-expressed c-Kit^+^CD41^+^ (2.26% ± 0.34) and c-Kit^−^CD41^+^ cells (4.05% ± 0.49) were also observed in ORI + IR vs IR group (c-Kit^+^CD41^+^: 1.67% ± 0.24 and c-Kit^−^CD41^+^: 2.93% ± 1.02). At day 18, the differences were shifted from immature c-Kit^+^CD41^−^ towards co-expressed c-Kit^+^CD41^+^ (5.38% ± 0.36) and mature c-Kit^−^CD41^+^ (4.51% ± 0.25) cells in ORI + IR group while in IR group shift was visible only in c-Kit^+^CD41^+^ fraction (3.61% ± 0.35) but not in c-Kit^−^CD41^+^ fraction (2.96% ± 0.36). These progenitor populations exhibited a moderate increase in Orientin alone group compared to unirradiated CT group at the tested time points (data not shown). The differences noted in fractional analyses of hematopoietic surface marker and CD41^+^ cells suggested that Orientin triggers MK cell formation and maturation at different stages of megakaryopoiesis.

Analysis of MK surface marker CD41 and its co-expression with activated platelet marker CD62P at day 9 and 18 exhibited a noticeable sustained increase of 1.48 fold in CD41^+^ cells (*P* < 0.05) and 1.3 fold in CD41^+^CD62P^+^ cells (*P* = 0.002) at both time points in ORI + IR compared to IR group (Figure S2b). The activated platelets (CD41^−^CD62P^+^) were almost undetectable at day 9 and upregulated by day 18 in ORI + IR and IR groups without any sharp difference between the groups. The specificity of Orientin towards MK cells was further validated by another set of experiments where BM cells were stained for erythrocyte surface marker Ter119 on day 18. An increase in erythroid cells was recorded in all irradiated mice compared to unirradiated mice groups, however the percentage of these cells was dropped in ORI + IR (41%) vs IR (47%) (Figure S2c). Furthermore, we have not seen any difference in basal Ter119^+^ cell ratio between unirradiated controls and Orientin alone cohorts suggested that altered erythroid population in ORI + IR vs IR could be a result of homeostatic balancing, triggered by Orientin post radiation injury.

### Orientin triggers megakaryocyte-specific transcriptional program

**Figure 4:**
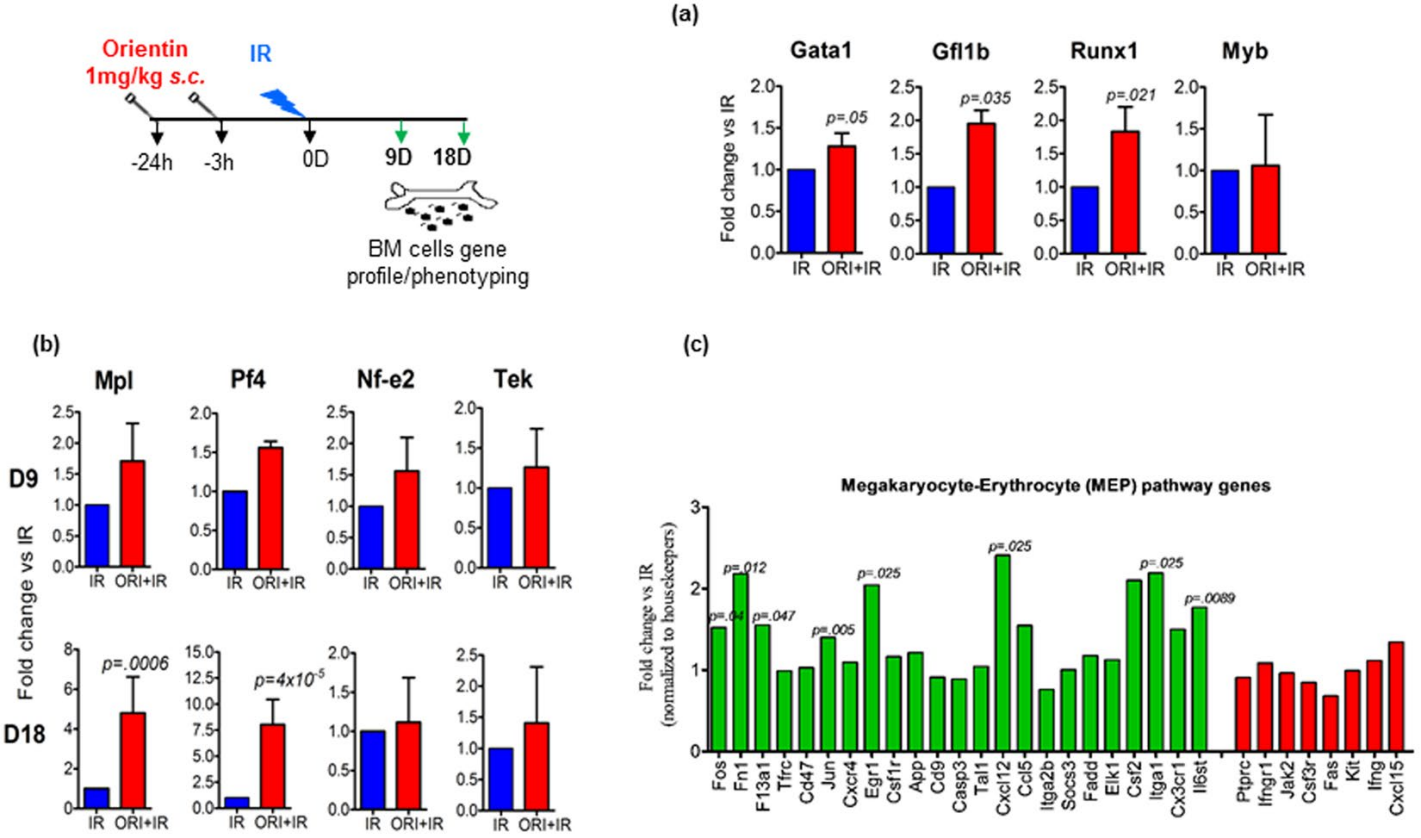
Real-Time PCR and NanoString analysis showing stimulatory response of Orientin on MEP transcriptional factors and key regulators of megakaryopoiesis. RNA isolated from mouse bone marrow mononuclear cells was used for Real Time quantitative RT-PCR analysis of (**a**) transcriptional factors of megakaryopoiesis at day 9 after irradiation and (**b**) associated genes at day 9 and 18 and (**c**) NanoString analysis of RNA from mouse BM mononuclear cells using genes from mouse inflammation panel at day 9. Histograms show fold change in ORI + IR vs IR. The megakaryocyte genes are in green and erythroid genes in red. Statistical significance was calculated using a Student *t-*test. For NanoString data, two-way ANOVA was performed for statistical significance between IR and ORI + IR group (n = 3 each).

In order to get mechanistic insight into the differences noted in the mature megakaryocytes and platelets, we investigated selected key transcriptional factors regulating megakaryopoiesis such as Gata1, Gfi1b, Runx1, and Myb by real-time PCR method on day 9 BM cells. While no major changes were observed in Myb (1.08 fold) expression, a significant increase was found in Gata1 (1.3 fold; *P* = 0.05), Gfi1b (1.74 fold; *P* = 0.035) and Runx1 (1.8 fold; *P* = 0.021) mRNA expression in ORI + IR group compared to IR alone group (Fig. 4a). These genes were also upregulated in mice treated with Orientin alone vs CT (data not shown). Comparison of genes involved in megakaryopoiesis on day 9 and 18, showed a 1.7 fold increase in expression of Mpl (TPO binding receptor) in ORI + IR vs IR at day 9, which further increased to 4.8 fold at day 18 (*P* = 0.0006) (Fig. 4b). Furthermore, Orientin administration significantly upregulated the expression of platelet factor Pf4 (Cxcl4) from 1.56 fold at day 9 to almost 8 fold at day 18 in ORI + IR mice vs IR alone (*P* = 0.00004). In addition, we observed a significant (1.5 and 1.3 fold) increase in Nf-e2 and Tek respectively in ORI + IR at day 9.

We further compared changes in expression of several of the gene transcripts that are associated with upstream and downstream of hematopoiesis available in a 749 gene panel in NanoString inflammation assay in BM cells on day 9. The ANOVA test shortlisted 446 genes with significant changes in expression compared to CT (unirradiated and untreated control group). Amongst the shortlisted genes, 6 genes > 2 fold, 16 genes > 1.5, 31 genes > 1.25 fold were found upregulated while 9 genes were < 0.5 fold down regulated (*P* < 0.05) along with 385 unaltered genes detected in ORI + IR compared with IR groups. Interestingly, the most significantly altered genes identified in ORI + IR groups were megakaryocyte specific Egr1, Cxcl12, IL6st, Fn1, clotting factor F13a1, Ccl5 and Csf2 genes^20–25^ (Fig. 4c). These molecules are commonly associated with development, differentiation, and expansion of megakaryocyte-erythroid progenitors.

### Orientin promotes bone marrow homeostasis by protecting the hematopoietic progenitors

Irradiation limits the clonogenic potential of stem cells; therefore, we were interested to find out whether or not Orientin has any effect on improving the hematopoietic progenitors in colony formation assay. Megakaryocytes were found increased in bone marrow after Orientin administration which led us to investigate the bone marrow stem cells for myeloid lineage colony formation. As expected, the colonies derived from mouse bone marrow stem cells harvested on day 9 post irradiation showed differences in the count. The colony assay using bone marrow stem cells harvested on day 9 post irradiation showed differences in the colony formation of progenitors. ORI + IR exhibited higher total number of colonies compared to IR alone (*P* = 0.0006) (Figure S3a), including multipotent progenitors CFU-GEMM (6.1 ± 3.3 vs 2.07 ± 1.17 in IR; *P* = 0.0.19) and CFU-GM (23.5 ± 5.17 vs 10.15 ± 5.76 in IR; *P* = 0.004), BFU-E (5.5 ± 2.2 vs 2.08 ± 1.18 in IR; *P* = ns) (Figure S3b). However, we did not notice any measurable differences in progenitor colonies between unirradiated controls and Orientin alone groups indicating that the effect of agent becomes apparent only under compromised bone marrow environment resulting from radiation injury.

### Orientin enhances megakaryocyte-erythrocyte lineage in human hematopoietic stem cells

**Figure 5:**
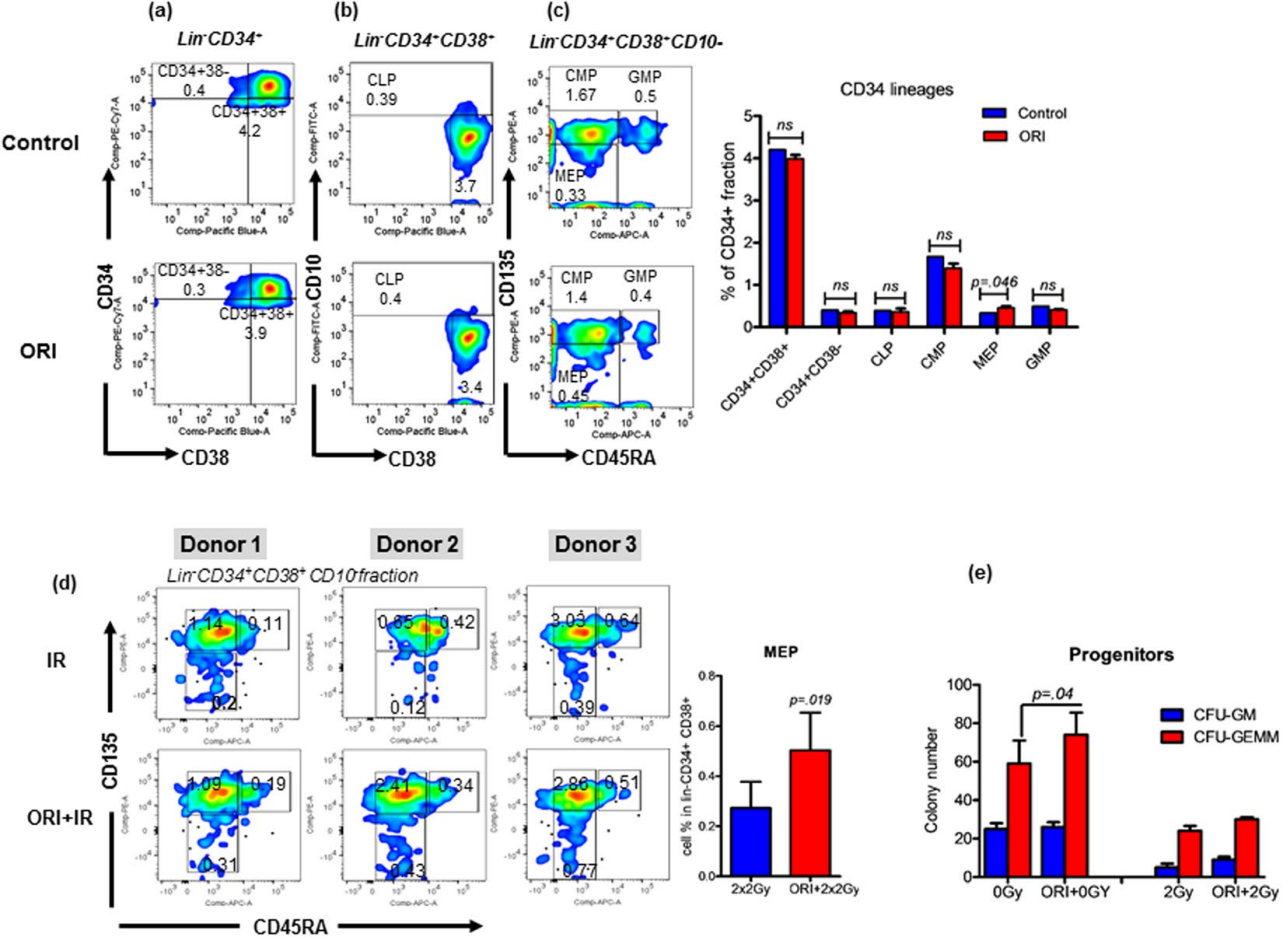
Effect of Orientin on human hematopoietic stem cells. (**a**–**c**) Orientin induces human CD34^+^ cells differentiation towards MEP lineage. CD34^+^ cells purified from healthy donor’s PBMCs via density gradient ficol followed by magnetic bead separation were treated with or without Orientin in a serum free expansion medium (SFEM) supplemented with 100 ng/ml cytokine cocktail (CC100) for 7 days. Cells were harvested for lineage differentiation and stained with CD34-PE-Cy7, CD38-Pacblue, CD10-FITC, CD135-PE, CD45RA-APC antibodies and subjected to FACS analysis. (d) Orientin partially restores CD34^+^ cells differentiation potential after irradiation. CD34^+^ cells purified from healthy donor’s PBMCs (1 × 10^5^/ml in triplicates) were treated with or without Orientin (5 µM) and exposed to 4 Gy (fractionated dose of 2 × 2 Gy). These cells were then co-cultured on human mesenchymal stromal cell layer in SFEM medium supplemented with 100 ng/ml cytokine cocktail for normal differentiation in 7 day time period. The MEP cells were gated in lin^−^CD34^+^CD38^+^CD10^−^CD135^−^CD45RA^−^ block in each sample. (**e**) Colony Assay in CD34^+^ cells (1 × 10^3^/ml in triplicates) post 0 or 2 Gy X-ray exposure. CFU-GM and GEMM colonies were scored on day 14 by applying standard morphologic criteria.

To further investigate the translational utility of Orientin, we evaluated its effects *ex vivo* in purified CD34+ human hematopoietic stem cells. These cells were purified from normal human PBMCs and were treated with various concentrations of Orientin (0.1 µM to 5 µM) in cell culture medium. First, we checked whether or not the agent has any effect on cell viability by Annexin assay on irradiated cells (4 Gy). A significant increase in live cells percentage (Annexin-PI- 35%, *P* = 0.01) was observed in Orientin (5 µM) pretreated cells compared with irradiated alone cells (28%). However, at lower concentration (0.1 and 1 µM), the effect was less prominent (Figure S4). We further examined the lineage differentiation potential of unirradiated CD34^+^ cells in presence or absence of Orientin for 7 days. Cells were harvested and stained for lin^−^CD34^+^CD38^+^ compartments common lymphoid progenitors (CLP; lin^−^CD34^+^CD38^+^CD10^+^), common myeloid progenitors (CMP; lin^−^CD34^+^CD38^+^CD10^−^CD135^+^CD45RA^−^) (Fig. 5a), granulocyte monocyte progenitors (GMP; lin^−^CD34^+^CD38^+^CD10^−^CD135^+^CD45RA^+^) (Fig. 5b) and megakaryocyte erythroid progenitors (MEP; lin^−^CD34^+^CD38^+^CD10^−^CD135^−^CD45RA^−^) (Fig. 5c), as described earlier^26,27^. Population segregation demonstrated a noticeable effect of Orientin on MEPs (1.45 fold compared to irradiated alone; *P* = 0.05) but not on other lineages. We also observed a significant increase in MEP population in CD34^+^ cells pre-stimulated with Orientin and exposed with a fractionated clinically relevant dose of 2 × 2 Gy X-rays vs untreated cells (*P* = 0.019) (Fig. 5d). In colony formation assay, Orientin pretreated and unirradiated CD34^+^ cells formed higher numbers of CFU-GEMM (70 ± 11.5/1 × 10^3^) compared to untreated and unirradiated group (55 ± 12/1 × 10^3^ CD34^+^; *P* = 0.04) with similar number of CFU-GM colonies. However, after irradiation due to the likely loss of a major stem cell fraction, we could not record much of the difference between IR vs ORI + IR cells. As expected, there was a marked decrease of up to 64% in overall colony forming units at 2 Gy irradiated CD34^+^ cells compared to Sham (0 Gy) irradiated cells where Orientin treatment could moderately rescue both GM and GEMM (Fig. 5e). Overall, multiple lines of evidence support that Orientin has the ability to augment HSC differentiation towards MEP lineage, resulting in increased MK cells and circulating platelets.

## Discussion

Longitudinal analyses of blood parameters in a pre-clinical animal model allowed discovery of a novel beneficial effect of a flavonoid glycoside, Orientin that is reported to be present in some tropical plants^28^. Importantly, the agent was found effective at a lower dose, physiologically achievable by dietary supplementation of Tulsi leaves. The efficacy at lower concentration indicate that the effect is not simply an antioxidant effect, an earlier reported mechanism of action of Orientin^12^. The data shows that platelet recovery by Orientin is attributed to the activation of MEP specific gene expression pathways. Gene expression analysis showed that the effect is possibly through modulation of various genes associated with development and proliferation of MK cells. Changes were noted in several of megakaryopoiesis transcription factors, including Gfi1b, Gata1 and Runx1 that are among transcription factors known to regulate post commitment step of MEP pathway^29–31^. They also serve multiple downstream functions such as MK maturation and polyploidization to produce proplatelets^32–34^. Runx1 in conjunction with Gfi1b ensures the maturation of megakaryocytes^10^. The upregulation of Mpl, Pf4, Nf-e2 and Tek/Tie2 in ORI + IR mice could be due to enhanced activity of transcriptional factors promoting megakaryopoiesis. The binding of Mpl receptor with its ligand Tpo is a hallmark of megakaryopoiesis and activates several signaling pathways, including RAS/MAPK and JAK-STAT, which lead to MK endomitosis and maturation^35–38^. The increased Mpl activity noted suggest that Orientin induces expansion of Mpl expressing stem cells and early megakaryocyte progenitors in bone marrow. Moreover, the regulation of Tek/Tie2 is associated with Mpl signaling in long term HSC expansion^35^. Pf4 is specifically involved in the maturation step of MK cells which is coordinated by Nf-e2. The absence of Nf-e2 is reported to be associated with lack of platelets in circulation^39^.

Orientin and Tulsi are likely conferring protection in MK cells and their location in bone marrow both at steady state and after radiation injury and to some extent helps in maintenance of hematopoietic stem cells self-renewal, proliferation, and differentiation. Agent seemingly impacts the mobilization of platelets from marrow to circulation in irradiated mice as evident from modulated expression of Cxcl12/Cxcr4. This molecular axis is a key regulator of maturational chemotaxis of MK cells toward sinusoidal vessels and also leads to MK formation in Tpo independent manner^40^. After bone marrow injury with cytotoxic agents, Cxcl12 gradient largely defines the fate of platelet release from MK and correlates with MK niche occupancy. The effect of Orientin on human PB CD34^+^ cells to differentiate in megakaryocytes indicates a stimulatory function in a relatively more stable microenvironment with released chemokines and cytokines.

Thrombocytopenia and platelet imbalances are reported in over 30% hematological admissions in hospitals, and a significant percentage of these patients who need treatment, including platelet transfusion to prevent bleeding. Supplementation of basils or other Orientin containing plant extracts will likely help increasing platelet output in patients receiving systemic therapy and other diseases resulting consequential platelet decrease in peripheral blood. Thrombocytopenia is also a major problem in mosquito-borne viral diseases such as dengue fever. Dietary administration of Orientin containing basils could be a viable and economic approach benefiting such patients in the developing world.

## Materials and Methods

### Animal experiments

Animal studies were carried out in compliance with guidelines approved by Institutional Animal Care and Use Committee (IACUC) of The Ohio State University (OSU) under the protocol 2011A00000029-R1.

### Irradiation and treatment plan/agent administration

C57BL/6 male mice (8–10 weeks old) were purchased from Charles River laboratory and housed in ULAR facility of The Ohio State University. Animals were randomized and divided in four groups Irradiated control (IR), and Orientin + irradiated (ORI + IR), PBS injected + unirradiated (CT) mice, and Orientin alone treated mice (ORI). Additional CT and ORI were used also to evaluate Orientin specific effects without radiation. Unanesthesized mice were exposed to 6 Gy TBI at a dose rate of 0.9 Gy/min using Gammacell®40 Exactor (Source ^137^Cs). Orientin (#QP-430, Quality Phytochemicals or #O9765, Sigma) was dissolved in PBS and was administered subcutaneously at 1 mg/kg body weight 24 h and 3 h pre or post irradiation while untreated control animals received PBS alone (supplemental Table 3). Experiments were repeated three times each with at least five animals in each group. For administration of Basil leaves, mice were fed ad libitum with Teklad LM-485 Powdered Rodent Diet (Envigo, with 44.3% carbohydrate, 19.1% protein, 5.8% fat) mixed with or without (100:1 ratio by weight) dried dietary Tulsi leaf powder. C57BL/6 male (8–10 weeks old) mice (N = 30) were evenly assorted to one of five different feeding groups: 10 mg Tulsi/1 g standard diet (Tulsi), 10 mg Tulsi/1 g standard diet + irradiation (Tulsi + IR), irradiation + 10 mg Tulsi/1 g standard diet (IR + Tulsi) and standard diet + irradiation (IR) and controls (supplemental Table 4). Powdered diets were administered via feed jars 3 days prior to irradiation and continuously until 14 days post irradiation or as specified. Food consumption was measured daily.

### Blood collection

Blood samples (about 50 µl) were collected, without sedation or anesthesia by submandibular bleed into a vacutainer containing EDTA as an anti-coagulant (Microvette® 100K3E SARSTEDT, #20.1278.100, Germany). Sampling was done from CT, IR, ORI + IR and IR + ORI treated mice groups at day 0, 1, 4, 7, 14, 17, 21, 24, and 30 with reference as radiation exposure for hematology. Two separate batches of mice were used to minimize stress due to repeated bleeding. Blood samples from Tulsi treated or untreated control mice were collected at day 10 for CBC while from irradiated group mice, blood was collected at day 14 and 21 for flow cytometry analysis. Blood samples were immediately processed on an automated CBC analyzer at Clinical Histology/Immunohistochemistry Laboratory within the College of Veterinary Medicine, The Ohio State University.

### Histology

Mice were euthanized by carbon dioxide inhalation followed by cervical dislocation as per standard operating procedure approved by IACUC of The Ohio State University. Femurs were collected, fixed in formalin for at least 24 h followed by decalcification for another 48 h in Leica Surgipath decalcifier. These bones were then sectioned longitudinally for H&E staining. The megakaryocytes identified by their characteristic shape of a large lobulated nucleus^41^, were counted in an area of 2.5 mm^2^ with the use of Image Scope v12.2.2.5015 (www.aperio.com) and confirmed by board certified histopathologist. In brief, quantification commenced 125 µm from the growth plate and an area of 2500 µm was scored.

### Bone marrow and c-kit^+^ cells isolation

Total BM cells were obtained by flushing the cavity of femurs, tibias, and ilium and filtered through 40 um filter. Mononuclear cells were collected by density gradient centrifugation using Ficoll-Paque Plus (#17-1440-02, GE healthcare). Buffy coat was then transferred to fresh tube and washed twice with sterile PBS. BM-MNCs were further depleted for c-Kit^+^ cells isolation using CD117 microbeads, mouse (#130-091-224, MiltenyiBiotec) as described earlier^42^.

### Methylcellulose assay

The evaluation of HSCs progenitor potential in treated groups bone marrow c-Kit+ cells were sorted and seeded in methylcellulose medium (#H4034, Stem Cell Technologies) at a concentration of 1 × 10^3^ cells /ml in triplicates. Numbers of colonies were counted after 7 days and scored by standard morphologic criteria.

### Flow cytometry

BM cells were flushed out of femurs and tibias of each group mice at both time points and further depleted to remove RBCs using RBC lysis buffer. The obtained pellet was stained with PE conjugated anti-mouse CD41 (clone MWReg30 #561850), FITC conjugated anti-mouse CD62P (#561923) for megakaryocytes and platelets and PE conjugated anti-mouse Ter119 (clone Ter119#561071). For stem cells analysis, cells were stained with APC conjugated anti-mouse CD117 (clone 2B8, #561074), and FITC-conjugated anti-mouse Sca1 (clone E13-161.7, #561077) antibodies (BD Biosciences). Flow cytometry was performed on LSR-II flow cytometer, and data was analyzed using Flow Jo v.10 or FSC Express 6 Plus deNovo softwares.

### Quantitative reverse transcriptase PCR and NanoString analysis

RNA was extracted from whole bone marrow cells using Ambion miRVANA kit (#AM1561, Life Technologies) and cDNA was prepared using High Capacity cDNA Reverse Transcription Kit (#4368813, Applied Biosystems). Real-time PCR was performed for megakaryocyte-erythrocyte specific genes (Gata1 #Mm01352636_m1, Pf4 #Mm00451315_g1, Mpl #Mm00440310_m1, Myb #Mm00501741_m1, Tek #Mm01256904_m1, Nf-e2 #Mm00801891_m1, Gflb1 #Mm00492318_m1 and Runx1 #Mm01213404_m1 using TaqMan primer probes (Thermo Fisher Scientific) on ABI-7900 detection system. Expressions were normalized to Gapdh/18S rRNA. An amplification-free, hybridization based direct digital counting method developed by NanoString Technologies was also applied to detect the changes in overall gene patterns using mouse cancer immune panel in Genomics Shared Resource (GSR), OSU. Raw data thus obtained from NanoString assays were first technically normalized followed by biological normalization with a set of 20 housekeeping genes (GEO accession no. GSE1000090).

### Human CD34^+^ cells isolation, differentiation and colony formation assay

Human blood samples were obtained from healthy donors (American RedCross Association). CD34^+^ cells were purified from PBMCs using Diamond CD34 isolation kit for human (#130-094-531, MiltenyiBiotec) as described by manufacturer. Purified CD34^+^ cells were cultured at 1 × 10^5^/ml in serum free SFEM medium (#09650, Stem Cell Technologies Inc.) supplemented with 100 ng/ml cytokine cocktail (CC100, Stem Cell Technologies Inc.) and stimulated with Orientin (1–5 µM) for 24 h and 3 h before 0, 2 or 4 Gy irradiation (X-rays using RS2000 Biological irradiator, RadSource) at a dose rate of 1.13 Gy/min. After irradiation, cells were co-cultured on human mesenchymal stromal cell layer to provide a microenvironment support for expansion and differentiation. All culture conditions were maintained at 37 °C in 5% CO_2_ for 7 days. After indicated period, cells were harvested, washed and stained with BD Biosciences primary antibodies of anti-human CD34-PE-Cy7 (clone 581#560710), CD38-Pacblue (clone HIT_2_#561378), CD10-FITC (#340925), CD135-PE (#558996), and CD45RA-APC (clone HI100#561884) for HSC lineages and CD41-PE (clone HIP8#560979) for MK cells analysis by flow cytometry. For colony assay, 1 × 10^3^ CD34^+^ cells from different treatment conditions were seeded in triplicates in methyl semi solid medium supplemented with cocktail of FBS, BSA, β-2ME, SCF, IL-3, EPO and GM-CSF (MethoCult-H4434, Stem Cell Technologies Inc. Canada). After 14 days, colonies were scored by standard morphologic criteria.

### Apoptosis assay

Apoptosis detection was done by Annexin-V assay (#556547, BD Biosciences) in human CD34^+^ cells were exposed to 4 Gy X-ray or Orientin (0.1–5 µM) + 4 Gy.

### Statistical analysis

Statistical significance was assessed by unpaired two tailed Student’s *t*-test. For NanoString data one way analysis of variance (ANOVA) was applied between the multiple treatment groups. Means with SD are reported wherever applicable. *P* values of < 0.05 were considered significant.

## Electronic supplementary material

Supplementary information

Supplementary data set
