# Supplementary material for: Ocimum flavone Orientin as a countermeasure for thrombocytopenia
Source: Sci Rep. 2018 Mar 22;8:5075. doi: 10.1038/s41598-018-23419-x (PMC5864743; doi:10.1038/s41598-018-23419-x)
Supplement: Supplementary file 1 — Supplementary information [file 41598_2018_23419_MOESM1_ESM.pdf]

Ocimum flavone Orientin as a countermeasure for thrombocytopenia

Marshleen Yadav, Feifei Song, Jason Huang, Arnab Chakravarti, Naduparambil K. Jacob*

Department of Radiation Oncology,

The Ohio State University Comprehensive Cancer Center

Columbus, OH 43210

*Correspondence: Naduparambil K. Jacob, PhD

Department of Radiation Oncology

Comprehensive Cancer Center

The Ohio State University

Columbus, OH, USA

Email: Naduparambil.Jacob@osumc.edu

Phone: 614-685-4246

Fax: 614-366-1938

Supplementary Figure Legends

Supplementary Figure S1: (a) Plateletcrit (PCT) measurement in Orientin pre and post IR treatment in mice exhibited a noticeable increase at day 17 and 21 compared with IR alone. Dot plot shows relative levels in comparison to irradiated animals. Statistical significance was calculated using a Student *t*-test. **(b)** Lymphoid and myeloid cells in mice peripheral blood post 6 weeks of sub-lethal irradiation. Cells were stained with primary antibodies of anti mouse-Ly6G, Mac1, CD3 and B220 for flow cytometry. Bar shows cell proportion compared to irradiated group and expressed as mean ±SD of 4 mice.

Supplementary Figure S2: Megakaryocytes surface protein CD41 expression in Orientin treated and irradiated mice. Flow cytometry of bone marrow cells at day 9 and 18 post irradiation. Co-expression of megakaryocyte surface marker CD41 with stem cells marker c-Kit^{+} **(a)** and platelet marker CD62P **(b)**. Erythroid cells (Ter119^{+}) **(c)**. Mononuclear cells were isolated from mouse whole bone marrow and stained with anti-mouse primary antibodies of CD41-PE, CD117-APC, CD62P-FITC and Ter119-PE. Bar shows relative expression compared to vehicle treated and unirradiated controls expressed as mean ±SD of 6 mice. Statistical significance was calculated using a Student *t*-test.

Supplementary Figure S3: Colony assay in c-Kit^{+} cells purified from mouse bone marrow of different treatment groups at day 9 post irradiation. Lin^{-}c-Kit^{+}cells (2x10^{3}cells/ml) were cultured in triplicates on mouse methylcellulose semi-solid medium supplemented with cytokine cocktail. CFU-GM, BFU-E and CFU-GEMM colonies were scored on day 7 by standard morphologic criteria. Bar shows average colony number generated from stem cells and compared to control untreated and unirradiated controls expressed as mean ±SD of 4 mice.

Supplementary Figure S4: Effect of Orientin on apoptosis in irradiated human hematopoietic stem cells ($CD34^+$). $CD34^+$ cells purified from healthy donor's PBMCs (n=3) via Ficoll-Paque density gradient method followed by magnetic bead sorting. Cells were treated with or without Orientin in a serum free expansion medium (SFEM) supplemented with 100ng/ml cytokine cocktail (CC100) were treated with various concentrations of Orientin (0.1to 5μM) 24 h before 4 Gy irradiation (X-ray). Cells were harvested after 24 h, washed and evaluated for live cell fractions and apoptotic cells by FACS using Annexin V staining.

Supplementary Figure S1

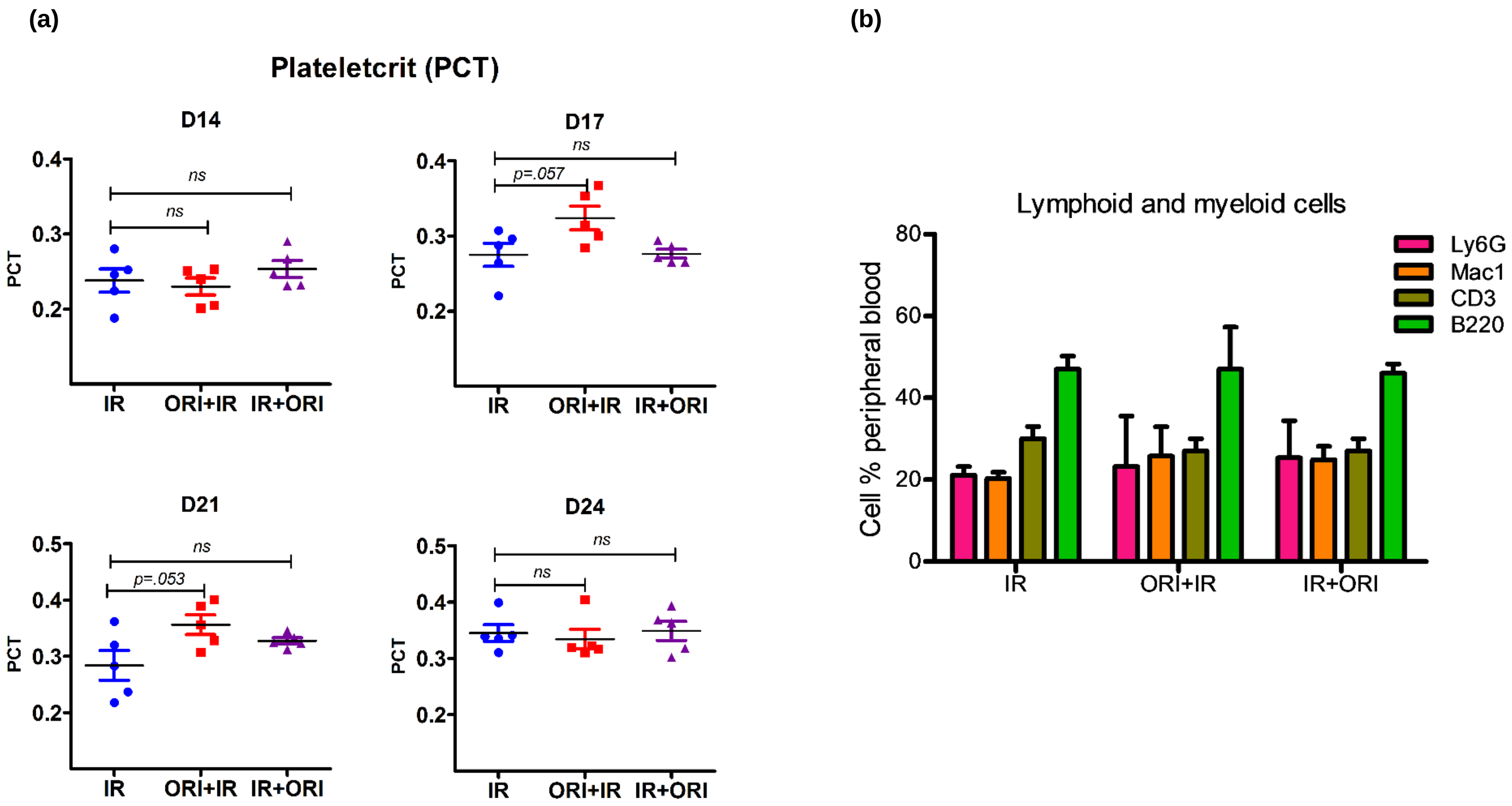

Supplementary Figure S2

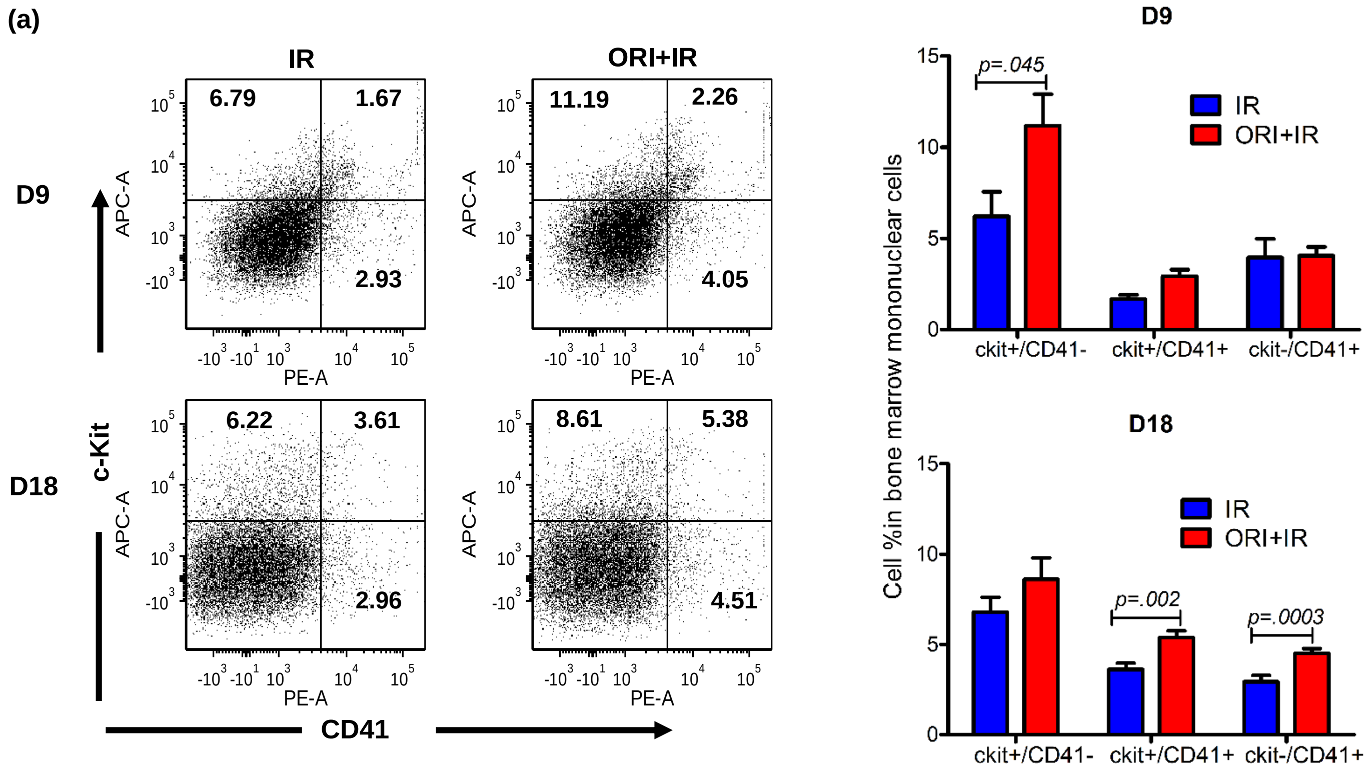

Supplementary Figure S2 contd.

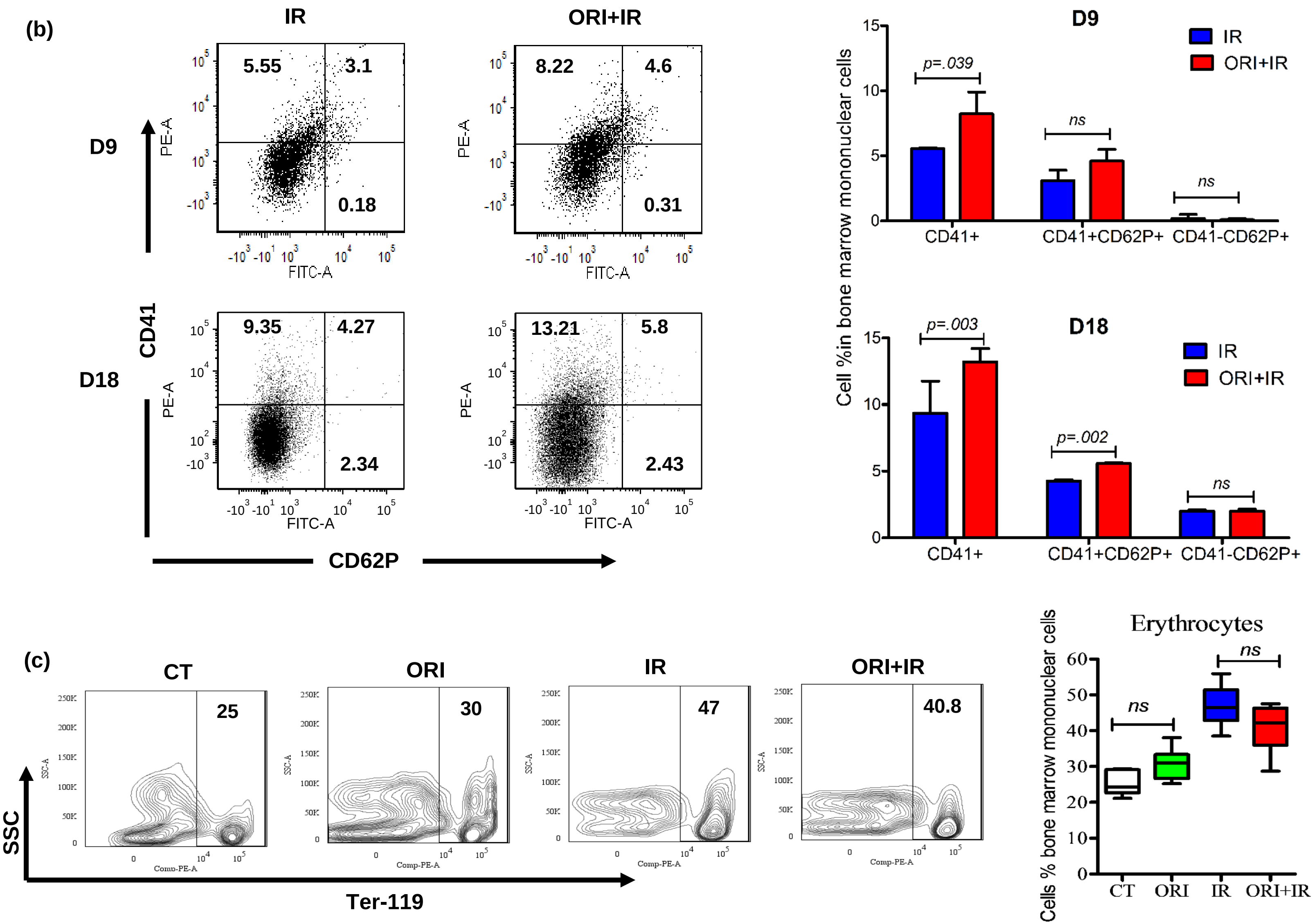

Supplementary Figure S3

(a)

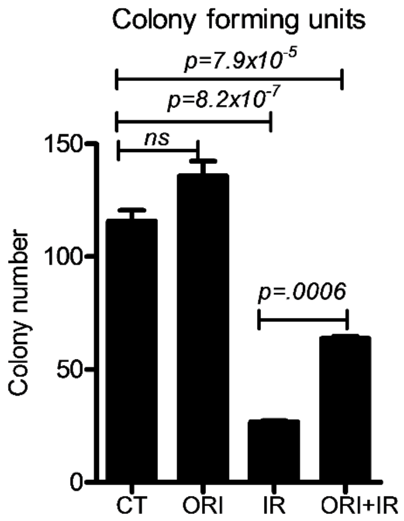

(b)

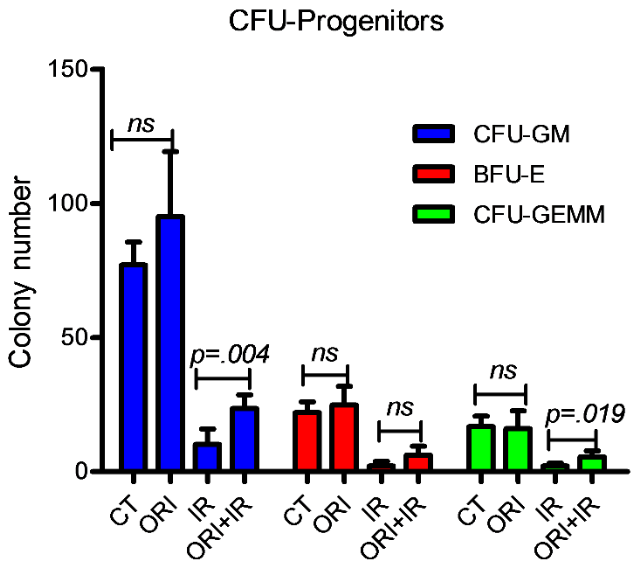

Supplementary Figure S4

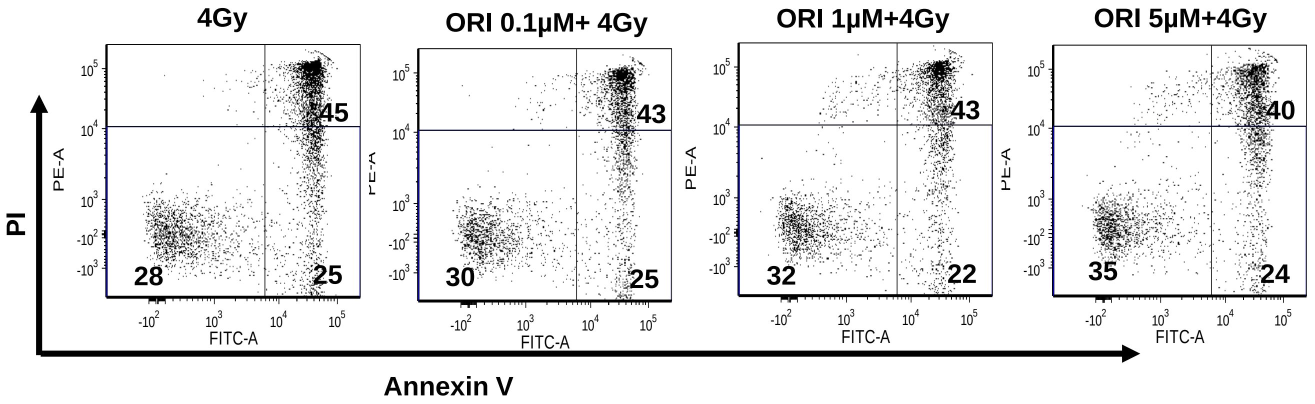

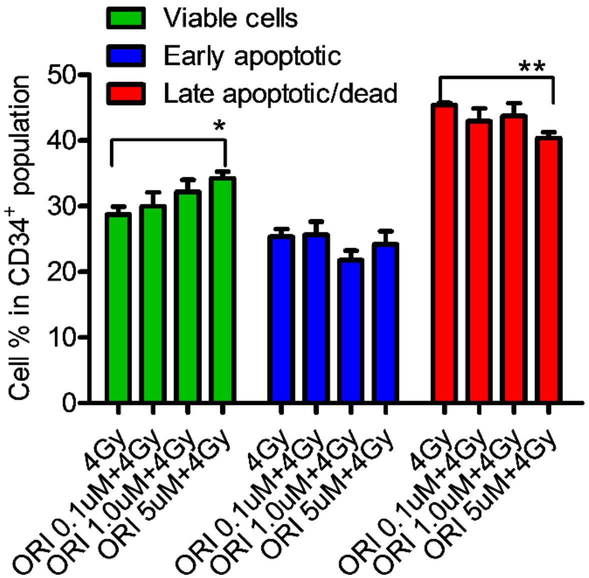

Supplementary Table 3: Orientin and radiation doses

**Group**	**Dose**	**Injections**
Control (CT)	PBS alone	Subcutaneous, twice, 24h and 3h before Sham (0Gy)
Orientin alone (ORI)	1mg/kg	Subcutaneous, twice, 24h and 3h before Sham (0Gy)
Irradiation alone (IR)	6Gy	Acute single dose, Total Body Irradiation (TBI) (^{137}Cs gamma rays)
Orientin + Irradiation (ORI+IR)	1mg/kg+6Gy (Pre)	Subcutaneous, twice, 24h and 3h before 6Gy TBI
Irradiation + Orientin (IR+ORI)	6Gy+1mg/kg (Post)	Subcutaneous, once, 3h and 24h after 6Gy TBI

Supplementary Table 4: Tulsi and radiation doses

**Group**	**Dose**
Control (CT)	standard powdered rodent chow, every day for 10 days
Tulsi alone (Tulsi)	10mg Tulsi/g standard diet (1%) ad libitum, every day for 10 days
Irradiation (IR)	6Gy acute single dose Total Body Irradiation (TBI)- ^{137}Cs Gamma rays
Tulsi + Irradiation (Tulsi + IR)	10mg Tulsi/g standard diet (1%), ad libitum for 14 days + 6Gy TBI (Pre)<br><br>Started three days before TBI
Irradiation + Tulsi (IR + Tulsi)	6Gy TBI + 10mg Tulsi/g standard diet (1%), ad libitum for 14 days (Post)<br><br>Started 3 hrs after TBI

Supplementary Table 5: List of mouse and human Flow cytometry antibodies

APC anti-Mouse CD117 (Clone 2B8)	#561074	BD Biosciences
Sca-1 FITC anti-Mouse Ly-6A/E (Clone E13-161.7)	#561077	
PE anti-Mouse CD41 (Clone MWReg30)	#561850	
FITC conjugated anti-mouse CD62P	#561923	
PE conjugated anti mouse Ter119	#561071	
PE-Cy-7 anti-human CD34 (clone 581)	#560710	
Pacblue anti-human CD38 (clone HIT$_2$)	#561378	
FITC anti-human CD10	#340925	
PE anti-human CD135-PE	#558996	
APC anti-human CD45RA(clone HI100)	#561884	
PE anti-human CD41-PE (clone HIP8)	#560979	
FITC Annexin V Apoptosis Detection Kit	#556547	

Supplementary Table 6: List of TaqMan probes

**TaqMan Probes**		**Company**
Gata1	#Mm01352636_m1	Thermo Fisher Scientific
Pf4	#Mm00451315_g1	
Mpl	#Mm00440310_m1	
Myb	#Mm00501741_m1	
Tek	#Mm01256904_m1	
Nf-e2	#Mm00801891_m1	
Gflb1	#Mm00492318_m1	
Runx1	#Mm01213404_m1	
